# Data heterogeneity and algorithmic bias in AI-based antimicrobial resistance prediction: a systematic review and mitigation framework

**DOI:** 10.1186/s12911-026-03729-1

**Published:** 2026-07-29

**Authors:** Jema Kiazolu

**Affiliations:** Department of Public Health, Hasanuddin University, Makassar, Indonesia

**Keywords:** Antimicrobial resistance, Artificial intelligence, Machine learning, Data heterogeneity, Algorithmic bias, Systematic review, Predictive modeling

## Abstract

**Background:**

Antimicrobial resistance (AMR) represents one of the most critical global public health threats of the contemporary era, contributing to millions of deaths and substantial morbidity worldwide. Artificial intelligence and machine learning (AI/ML) have been increasingly applied to AMR detection, prediction, and management, demonstrating promising results in controlled research settings. However, clinical translation remains substantially constrained by fundamental methodological challenges, particularly data heterogeneity and algorithmic bias, whose extent and consequences are not yet adequately characterised.

**Methods:**

A systematic review with descriptive synthesis was conducted in accordance with the Preferred Reporting Items for Systematic Reviews and Meta-Analyses (PRISMA 2020) guidelines. Searches were performed across PubMed/MEDLINE, Embase, Scopus, Web of Science, IEEE Xplore, and the ACM Digital Library, covering studies published between January 2020 and April 2026. Eligible studies applied AI/ML approaches to AMR prediction using whole-genome sequencing (WGS), antimicrobial susceptibility testing (AST), electronic health records (EHRs), surveillance datasets, and spectral data. Data were extracted using a standardized charting form and synthesized using descriptive and thematic methods. No meta-analysis was conducted owing to substantial methodological heterogeneity across included studies.

**Results:**

A total of 68 studies published between 2020 and 2026 were included, of which 21 underwent detailed analysis. Studies demonstrated substantial heterogeneity across data modalities, patient populations, laboratory practices, and geographic settings. WGS-based approaches were most frequently represented, followed by AST- and EHR-based models. While many models achieved strong internal performance, generalizability across external settings remained limited. Key sources of heterogeneity included variability in data modalities, laboratory protocols, population composition, and temporal and geographic distribution. Major forms of bias identified included sampling bias, label inconsistency, structural confounding, and clinical context bias. Mitigation strategies demonstrated partial and context-dependent improvements.

**Conclusions:**

Data heterogeneity and algorithmic bias are the primary constraints on AI/ML-based AMR prediction, not algorithmic sophistication. The strong internal performance metrics reported across the literature do not reliably generalize to diverse populations, geographic settings, or clinical environments. Meaningful progress requires a deliberate shift in research priorities toward globally representative datasets, harmonized laboratory standards, mandatory external validation, and equitable model development. The proposed Heterogeneity Mitigation Framework offers a structured, evidence-grounded approach to these challenges; its empirical validation in diverse real-world settings is the most important next step for the field.

**Systematic review registration:**

Although this review was not prospectively registered in PROSPERO, it was conducted using predefined eligibility criteria, structured data extraction, and transparent reporting in accordance with PRISMA 2020 guidelines.

## Introduction

Antimicrobial resistance (AMR) has emerged as one of the most pressing global public health challenges of the 21st century. The widespread misuse and overuse of antimicrobial agents in human medicine, agriculture, and animal health have accelerated the emergence and dissemination of resistant pathogens, rendering infections that were once readily treatable increasingly difficult or impossible to manage. Recent global estimates indicate that AMR contributes substantially to both morbidity and mortality, with millions of deaths associated with resistant infections each year [1]. This burden is disproportionately concentrated in low- and middle-income countries (LMICs), where deficiencies in diagnostic capacity, surveillance infrastructure, and access to effective therapies further amplify adverse outcomes [2].

In response to the growing complexity of AMR, artificial intelligence (AI) and machine learning (ML) have increasingly been applied to enhance detection, prediction, and clinical management of resistance. These methods leverage large-scale biomedical datasets encompassing WGS, AST, EHRs, surveillance systems, and mass spectrometry to identify complex, non-linear associations with resistance phenotypes [3, 4]. Commonly employed models—including random forests, support vector machines, gradient boosting algorithms, and deep learning architectures—have demonstrated moderate to high predictive performance across a range of pathogens and clinical contexts [5, 6]. Beyond prediction, AI-driven tools are progressively being incorporated into clinical decision support systems and antimicrobial stewardship programs to guide empiric therapy selection and optimize antibiotic prescribing [7, 8].

Despite these advances, the clinical translation of AI/ML-based AMR prediction models remains substantially limited. A central challenge is data heterogeneity, arising from variability in data sources, formats, laboratory protocols, and patient populations. AMR datasets are inherently multi-modal, integrating genomic, phenotypic, clinical, and surveillance data that each differ in structure, quality, and completeness. Such variability complicates data integration, reduces reproducibility, and undermines model generalizability across settings [9, 10]. Closely related to heterogeneity is algorithmic bias, whereby models learn systematic distortions embedded in training data rather than true biological or clinical relationships. In healthcare applications, these biases can produce unequal model performance across subpopulations, potentially reinforcing existing health disparities [11, 12].

For instance, models trained predominantly on data from high-income settings may perform poorly in LMIC contexts, where resistance patterns, healthcare systems, and data infrastructures differ substantially [13, 14]. Furthermore, many AMR prediction models rely on single-center datasets and internal validation strategies, resulting in overestimated performance metrics and limited external validity when applied to new populations or clinical environments [4, 5]. These challenges expose a critical gap between methodological development and real-world implementation of AI in AMR prediction.

While existing studies have explored the application of ML to AMR prediction, a comprehensive synthesis focusing specifically on the interconnected roles of data heterogeneity and algorithmic bias remains lacking. In particular, the mechanisms through which heterogeneity generates bias, and how these factors jointly influence model robustness, fairness, and generalizability, have not been systematically characterized.

This study therefore aims to synthesize current evidence on data heterogeneity and algorithmic bias in AI/ML-based AMR prediction and to propose a structured framework for their mitigation. Existing reviews have catalogued algorithmic approaches and reported performance metrics across pathogen-antibiotic combinations, but none has systematically examined the mechanisms by which data heterogeneity generates and sustains algorithmic bias across the full modelling pipeline, nor why these challenges persist despite being well-recognised. This paper addresses that gap by characterising not merely what the problems are, but why they recur: the structural incentives, reporting norms, and data ecosystem conditions that cause the same limitations to appear across successive generations of AMR machine learning studies. By shifting focus from predictive performance alone to the structural and methodological conditions required for robustness, fairness, and real-world applicability, this work provides a diagnostic account of implementation failure alongside a framework for its remediation.

## Methods

### Study design

This study employed a systematic review design with descriptive synthesis, conducted in accordance with the PRISMA 2020 framework [15]. A systematic review approach was selected to provide structured, reproducible, and transparent identification and synthesis of evidence on data heterogeneity and algorithmic bias in AI-based AMR prediction. The review protocol emphasized comprehensive database searching, independent study selection, standardized data extraction, and thematic synthesis. No meta-analysis was conducted owing to substantial methodological and outcome heterogeneity across included studies, which precluded statistical pooling. This review was not prospectively registered in PROSPERO.

### Eligibility criteria

Eligibility criteria were defined using the Population–Concept–Context (PCC) framework to ensure structured and transparent study selection [16].

#### Inclusion criteria

Studies were included if they: (1) applied AI, ML, or deep learning (DL) methods to predict AMR-related outcomes, including susceptibility classification, minimum inhibitory concentration (MIC) prediction, or resistance risk estimation; (2) reported model development, validation strategies, or performance metrics such as accuracy, sensitivity, specificity, F1-score, or area under the curve (AUC); (3) utilized one or more data modalities including AST, WGS, EHRs, surveillance datasets, or spectral data such as MALDI-TOF mass spectrometry; (4) were conducted in clinical, laboratory, public health, or surveillance contexts; and (5) were primary research studies, conference proceedings, or review articles providing methodological or conceptual insights.

#### Exclusion criteria

Studies were excluded if they: (1) used non-AI approaches, including rule-based or traditional statistical models without machine learning components; (2) were purely experimental laboratory studies without predictive modeling; (3) did not report predictive outcomes or model performance; or (4) were editorials, commentaries, or opinion papers without methodological or empirical contribution. These criteria ensured that included studies contributed to both empirical evidence and conceptual understanding of heterogeneity, bias, and generalizability in AI-based AMR prediction.

### Information sources and database search

**Fig. 1:**
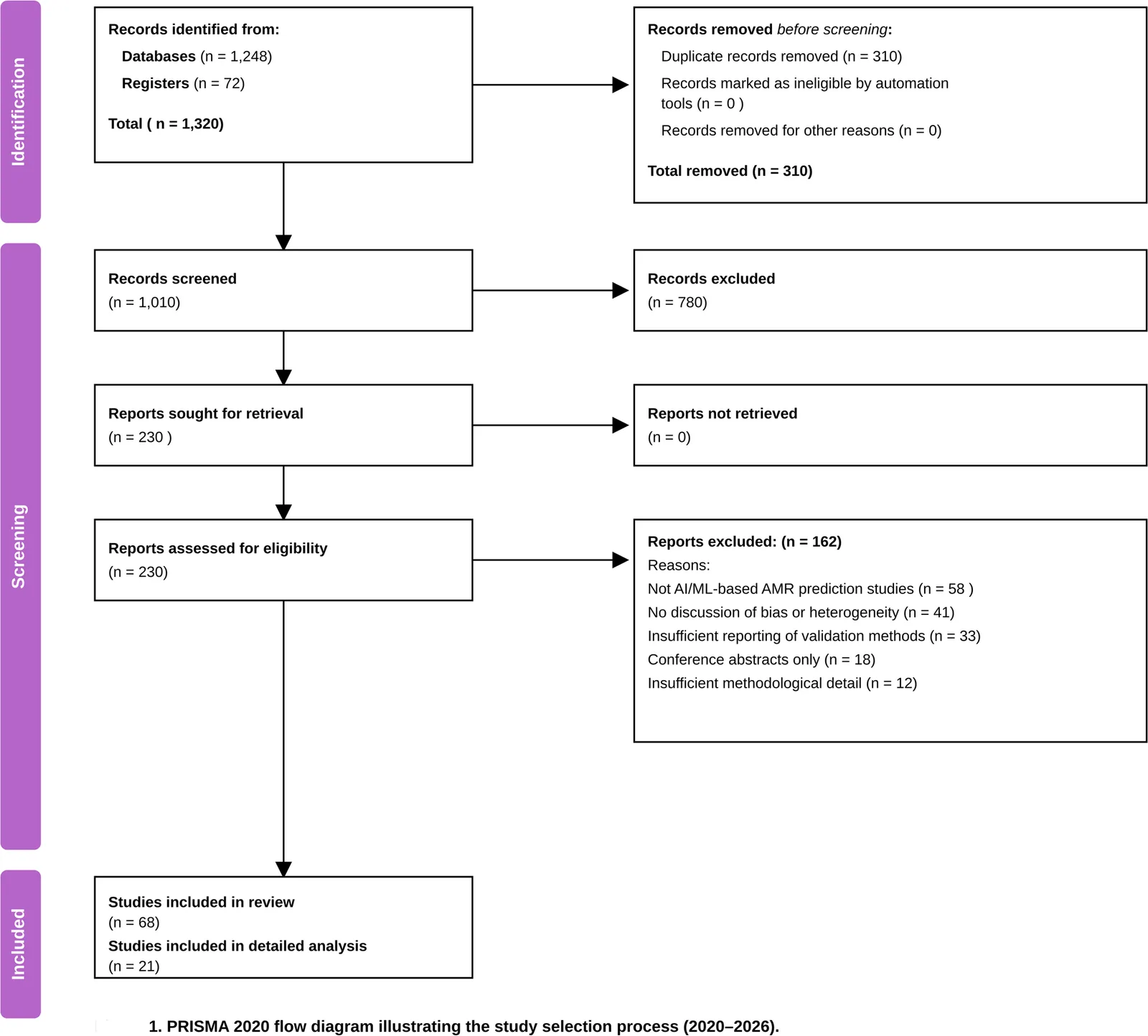
PRISMA 2020 flow diagram of study selection process

A comprehensive literature search was conducted across PubMed/MEDLINE, Embase, Scopus, Web of Science, IEEE Xplore, and the ACM Digital Library, to capture interdisciplinary research spanning biomedical, computational, and public health domains. The search was restricted to studies published between January 2020 and April 2026 to reflect recent methodological developments in AI and AMR prediction. To enhance comprehensiveness, reference lists of included studies and relevant reviews were manually screened, and database-specific indexing terms, including MeSH and Emtree, were adapted accordingly. The study selection process followed PRISMA 2020 reporting standards [15], including identification, screening, eligibility assessment, and inclusion of studies. The flow of study selection is illustrated in Fig. 1.

### Search strategy

The search strategy combined controlled vocabulary and free-text terms related to AI and AMR prediction. Key concepts included core terms (“artificial intelligence,” “machine learning,” “deep learning”), domain terms (“antimicrobial resistance,” “AMR,” “prediction,” “genomics,” “whole-genome sequencing”), and clinical terms (“antimicrobial susceptibility testing,” “surveillance,” “stewardship”). Boolean operators (AND, OR, NOT) were applied to optimize retrieval. Searches were restricted to studies published between January 2020 and April 2026.

### Study selection

All records were screened by a single reviewer (the author) in two stages: (1) title and abstract screening based on predefined eligibility criteria, and (2) full-text screening of potentially relevant studies. To ensure consistency and minimize bias, standardized inclusion and exclusion criteria were applied throughout the selection process.

### Data extraction

Data extraction was performed by a single reviewer (the author) using a standardized and piloted data extraction form to ensure consistency and completeness. Extracted variables included: pathogen and antibiotic focus; data modality (e.g., WGS, AST, EHR, surveillance); model type (e.g., random forest, support vector machine, neural networks); validation strategy (internal and/or external); performance metrics (e.g., accuracy, AUC, F1-score); sources of heterogeneity; types of bias; and reported mitigation strategies.

### Data synthesis

A combined descriptive and thematic synthesis approach was employed, consistent with established systematic review methodology for evidence that is unsuitable for meta-analysis [16]. Descriptive synthesis involved summarizing study characteristics using tables and visualizations, including modality distribution, model types, validation strategies, and geographic representation, to enable structured comparison across studies. Thematic synthesis involved a narrative approach to identify key patterns related to dimensions of data heterogeneity (e.g., modality, population, temporal variation), types of algorithmic bias (e.g., sampling bias, label bias, structural confounding), and strategies for their mitigation. Themes were developed inductively and refined through iterative reviewer discussion. Formal meta-analysis was not performed due to the substantial clinical, methodological, and statistical heterogeneity across included studies, which precluded meaningful pooling of effect estimates.

## Results

### Overview of included studies

A total of 68 studies published between 2020 and 2026 were included in this systematic review, of which 21 met the criteria for detailed analysis. These 21 studies were selected for in-depth analysis based on their relevance to data heterogeneity and algorithmic bias, as well as the completeness of methodological reporting. This reflects a growing yet methodologically heterogeneous body of literature on AI/ML-based AMR prediction. The characteristics of the 21 studies selected for detailed analysis are presented in Table 1.

**Table 1:** Characteristics of the 21 studies selected for detailed analysis in AI/ML-based amr prediction (2020–2026)

First Author (Year)	Study Type	Data Modality	Model Type	Pathogen Scope	Setting	Region
Khaledi (2020)	Primary	WGS	RF, SVM	P. aeruginosa	Hospital	Germany
Weis (2020)	Primary	MALDI-TOF	RF, CNN	Multiple	Clinical lab	Switzerland
Ren (2022)	Primary	WGS	RF, DNN	Multiple	Clinical isolates	Germany
Feretzakis (2021)	Primary	AST, Surveillance	Gradient Boosting	Multiple	ICU	Greece
Ardila (2024)	Primary	WGS + AST	RF, Ensemble	Priority pathogens	Healthcare	Colombia
Ferrari (2024)	Primary	EHR	LR, RF	Bloodstream infections	ICU	UK
Park (2024)	Primary	MALDI-TOF	CNN, RF	Multiple	Clinical lab	Germany
Cohen (2025)	Primary	EHR	ML, LLM	Sepsis	Hospital	USA
Valavarasu (2025)	Primary	Surveillance, AST	Time-series ML	Broad	Surveillance	India
Zou (2025)	Primary	WGS	CNN, DNN	Pediatric infections	Hospital	China
Urena (2024)	Primary	Clinical + Ecology	ML	Hospital infections	Hospital	France
Hardan (2024)	Primary	EHR (multimodal)	Neural Networks	Multiple	Hospital	Middle East
Blechman (2024)	Primary	EHR	Gradient Boosting	Broad infections	Hospital	USA
Condorelli (2024)	Primary	WGS	RF, XGBoost	K. pneumoniae	Clinical isolates	Italy
Zhao (2024)	Primary	Surveillance	ML	Food-animal pathogens	Surveillance	Global
Ardila (2025)	Primary	AST	RF, Gradient Boosting	Priority pathogens	Healthcare	Colombia
Nsubuga (2024)	Primary	WGS	ML	E. coli	Multicountry	Africa
Prosperi (2022)	Primary	Genomic	ML	Multiple	Mixed	USA
Sidorczuk (2022)	Primary	Peptide/Genomic	ML	Antimicrobial peptides	Computational	Europe
Kim (2022)	Review	WGS	—	Broad	Clinical	Canada/UK
Tang (2022)	Review	Multi-modal	—	Broad	Mixed	China

**Abbreviations**: WGS = Whole-genome sequencing; AST = Antimicrobial susceptibility testing; EHR = Electronic health records; RF = Random Forest; SVM = Support Vector Machine; CNN = Convolutional Neural Network; DNN = Deep Neural Network; LR = Logistic Regression

The included studies utilized diverse data modalities, underscoring the multi-domain nature of AMR prediction. These modalities included WGS, AST phenotypes, EHRs, spectral data (e.g., MALDI-TOF mass spectrometry), and surveillance datasets. WGS-based approaches were most frequently represented, followed by AST- and EHR-based models, while spectral and surveillance data were comparatively less common [17–19].

Geographically, the evidence base was heavily concentrated in high-income regions, particularly North America, Europe, and East Asia, where access to genomic sequencing infrastructure, clinical informatics systems, and large-scale surveillance data is more established [4, 20]. In contrast, representation from LMICs remained limited despite their disproportionate burden of AMR [13, 14].

Most studies were conducted in hospital-based settings, particularly among inpatient and intensive care unit (ICU) populations, where AMR prevalence is high and data availability is greater [7, 8]. Community-level and outpatient datasets were comparatively underrepresented. Although surveillance-based studies partially expanded geographic and population coverage, important gaps persisted in non-hospital and resource-limited settings [19]. Overall, the included studies demonstrate substantial heterogeneity across data modalities, geographic regions, and clinical settings, with a clear dominance of high-resource, hospital-based datasets. This imbalance has important implications for the generalizability and equity of AI/ML-based AMR prediction models.

### Dimensions of data heterogeneity

Data heterogeneity emerged as a central determinant of model performance, reproducibility, and generalizability. Heterogeneity was observed across multiple interacting dimensions, including data modality, laboratory practices, population characteristics, and temporal and geographic variation [5, 10, 20].

#### Data modality and measurement heterogeneity

Data modality heterogeneity arises from the integration of fundamentally different data sources, each capturing distinct aspects of AMR. WGS-based approaches depend on sequencing quality, bioinformatics pipelines, resistance gene databases, and genotype–phenotype mapping strategies, resulting in variability in feature representation and predictive outputs [17, 21]. AST-based models are influenced by methodological differences including broth microdilution, disk diffusion, and automated systems, as well as laboratory-specific protocols and class imbalance in resistance phenotypes [19, 22]. EHR-derived data introduce further heterogeneity through variability in coding systems, documentation practices, and missing data patterns, complicating integration with genomic and phenotypic data [23, 24]. Similarly, MALDI-TOF-based approaches are sensitive to instrument calibration, sample preparation, and spectral preprocessing, generating variability across laboratories [18, 25]. Collectively, these modality-specific differences create significant interoperability challenges and limit the development of unified, multi-modal prediction models.

#### Laboratory and technical heterogeneity

Laboratory protocols introduce systematic variability that directly affects resistance measurement and model performance. Differences in AST methodologies and interpretive breakpoints particularly between CLSI and EUCAST standards lead to inconsistent resistance classification across datasets [22, 26]. In WGS-based approaches, sequencing depth, quality control procedures, and bioinformatics pipelines further contribute to heterogeneity, resulting in discordant resistance gene detection and reduced predictive reliability [27]. These technical inconsistencies introduce measurement bias and undermine comparability across studies, particularly in multi-center datasets.

#### Population and setting heterogeneity

Population-level heterogeneity reflects imbalances in geographic, demographic, and clinical representation within datasets. Most studies were conducted in high-income countries, resulting in limited representation of LMICs where resistance patterns and healthcare systems differ substantially [4, 14]. Datasets were predominantly derived from hospital-based populations, while community and outpatient data were underrepresented [20]. Variability across patient groups including differences between pediatric and adult populations further complicates model generalizability. Imbalances in pathogen–antibiotic combinations contribute additionally, with overrepresentation of commonly studied species and underrepresentation of rare or context-specific resistance patterns [28].

#### Temporal and geographic heterogeneity

Temporal variation introduces complexity through changes in antimicrobial prescribing practices, resistance prevalence, and laboratory methodologies over time phenomena that can degrade model performance when applied to future datasets [9, 14]. Geographic heterogeneity further limits model transferability, as resistance patterns vary across regions due to differences in microbial ecology, healthcare systems, and surveillance infrastructure [13, 28]. These dynamics highlight the importance of context-aware model development and validation strategies.

#### Species and antibiotic imbalance

AMR datasets exhibit substantial imbalance across pathogen–antibiotic combinations, with frequently studied bacterial species and antimicrobial classes overrepresented while rare pathogens and certain antibiotic groups remain underrepresented [29]. Environmental and livestock surveillance data further reveal geographic biases, with disproportionate representation from specific regions [28]. This imbalance biases model training toward dominant patterns and reduces predictive performance for underrepresented species–drug combinations.

Across studies, data heterogeneity is multi-dimensional and interdependent, affecting data quality, interoperability, and model performance. These interacting dimensions reduce reproducibility, limit generalizability, and complicate cross-study comparability, thereby constraining the clinical applicability of AI/ML-based AMR prediction models [5, 6]. The key dimensions of data heterogeneity and their implications for AI/ML-based AMR prediction are summarized in Table 2.

**Table 2:** Key dimensions of data heterogeneity and their implications for AI/ML-based amr prediction

Dimension	Source of Heterogeneity	Mechanism	Impact on AI/ML Models
Data modality	WGS, AST, EHR, MALDI-TOF, surveillance data	Differences in data structure, format, and feature representation	Reduced interoperability; inconsistent feature learning
Laboratory practices	CLSI vs. EUCAST standards; AST methodologies	Variability in resistance measurement and classification	Label inconsistency; reduced reproducibility
Population & setting	High-income country dominance; hospital-based datasets	Sampling imbalance across populations and clinical settings	Poor generalizability; sampling bias
Temporal variation	Changing resistance patterns over time	Dataset shift and evolving epidemiological trends	Model performance degradation
Geographic variation	Regional differences in resistance patterns	Context-dependent resistance ecology	Limited transferability
Species–antibiotic imbalance	Uneven representation of pathogen–drug combinations	Skewed training distribution	Poor performance on underrepresented classes

### Types of algorithmic bias identified

While heterogeneity reflects variability in data, algorithmic bias represents the systematic distortions that arise from such variability during model development and deployment. Four major types of bias were consistently identified across studies.

#### Sampling bias

Sampling bias occurs when training datasets do not adequately represent the populations, geographic regions, or clinical settings in which models are subsequently applied, causing models to learn context-specific correlations rather than generalizable resistance mechanisms [13, 14]. Global datasets are often skewed toward high-income countries and hospital-based samples, limiting representation of LMIC contexts and community settings. Temporal shifts in resistance patterns further exacerbate sampling bias, resulting in performance degradation when models are applied to newer datasets [13, 30]. At the institutional level, models trained on single-center data often capture local prescribing practices and microbial ecology, leading to inflated internal performance but poor external validity [7, 8].

#### Label bias

Label bias arises from inconsistencies in resistance definitions and classification across datasets. Variability in susceptibility categories and MIC thresholds introduces systematic noise into training labels [26]. Differences between guideline systems such as CLSI and EUCAST can result in identical MIC values being classified differently, while aggregation of susceptibility categories may obscure clinically relevant distinctions. These inconsistencies reduce reproducibility and compromise model generalizability.

#### Structural (Confounding) bias

Structural bias occurs when models exploit non-causal correlations within the data, such as phylogenetic structure or species–drug co-occurrence patterns. Resistance phenotypes are often unevenly distributed across bacterial lineages, leading models to rely on lineage-specific markers as proxies for resistance rather than identifying causal mechanisms [21, 29]. Similarly, non-random pairing of pathogens and antibiotics in datasets introduces co-occurrence bias, where models learn dataset-specific relationships rather than biologically meaningful associations [5, 31].

#### Clinical context bias

Clinical context bias arises from artifacts embedded in EHR data, including healthcare access patterns, documentation practices, and missing data [23, 24]. Derived clinical variables and comorbidity indices may vary across populations, introducing additional inconsistencies [12]. Moreover, observational clinical data often reflect prescribing behaviours and healthcare system processes rather than causal relationships between antibiotic use and resistance [32]. These factors limit model transportability and raise concerns regarding fairness and reliability. The sources, mechanisms, consequences, and mitigation strategies of algorithmic bias identified across studies are summarized in Table 3.

**Table 3:** Sources, mechanisms, consequences, and mitigation of algorithmic bias in AI-based AMR prediction

Bias Type	Source	Mechanism	Consequence	Mitigation Strategies
Sampling bias	HIC-dominated datasets; single-center data	Non-representative training data	Poor external validity; limited generalizability	Multi-center datasets; reweighting; stratified sampling
Label bias	CLSI vs. EUCAST breakpoints; MIC variability	Inconsistent resistance classification	Misclassification; reduced reproducibility	Standardized breakpoints; use of raw MIC values
Structural bias	Phylogeny; species–drug co-occurrence	Learning non-causal correlations	Spurious predictions; reduced biological validity	Structure-aware modeling; phylogenetic splitting
Clinical context bias	EHR documentation; healthcare access differences	Context-dependent artifacts	Limited transportability; fairness concerns	Feature harmonization; bias auditing
Algorithmic bias	Class imbalance; subgroup disparities	Unequal representation in training	Unequal model performance	Resampling; cost-sensitive learning

### Mitigation strategies used in studies

Studies addressing AMR prediction using AI/ML employ a range of mitigation strategies targeting different stages of the modeling pipeline. These strategies can be broadly categorized into data-level, model-level, and evaluation-level approaches, as well as emerging explainability and causal methods. While these approaches contribute to improved robustness and fairness, their effectiveness remains partial and context-dependent, reflecting the underlying complexity of AMR data [6, 11].

#### Data-Level strategies: addressing class imbalance and sampling bias

Class imbalance is a pervasive challenge in AMR datasets, where resistant phenotypes or minority classes are frequently underrepresented. Common approaches include oversampling, undersampling, and synthetic data generation techniques such as the Synthetic Minority Over-sampling Technique (SMOTE), alongside cost-sensitive learning and weighted loss functions [33]. Resampling and reweighting techniques are widely applied in clinical and EHR-based models to improve representation of minority classes; however, their impact on overall performance is inconsistent and may introduce overfitting in small or highly imbalanced datasets [6]. Bias-aware sampling strategies have also been used to address imbalances across geographic regions, temporal distributions, and pathogen–antibiotic combinations, though reported improvements are generally modest and highly dependent on dataset characteristics [6, 29].

#### Structure-Aware modeling and data splitting

To mitigate structural and phylogenetic bias, several studies emphasize population structure-aware modeling and evaluation. Conventional random train–test splits allow models to exploit lineage-specific or dataset-specific correlations, leading to inflated performance estimates. In contrast, structure-aware approaches including phylogeny-informed data splitting, clade-based partitioning, and stratified sampling aim to ensure independence between training and testing datasets [21, 29]. These methods reduce reliance on non-causal correlations and promote the learning of more generalizable resistance mechanisms. However, they often result in lower reported performance metrics, reflecting a more realistic assessment of model capability.

#### Robust evaluation strategies

Robust evaluation is a critical component of bias mitigation, directly influencing the reliability and generalizability of model performance estimates. Key strategies include external validation using independent datasets, temporal validation across different time periods, and multi-center study designs [11]. Despite their importance, these approaches remain underutilized in the AMR prediction literature. Evidence consistently demonstrates that models evaluated on external datasets exhibit reduced performance compared to internal validation, indicating substantial overfitting to training data [6]. Beyond discrimination metrics such as AUC, there is increasing emphasis on reporting calibration, subgroup performance, and fairness-related metrics to provide a more comprehensive assessment of real-world applicability across diverse populations.

#### Explainability and causal approaches

Explainability techniques are increasingly incorporated to identify and mitigate bias in AI/ML models. Methods such as feature attribution, model interpretability tools, and fairness metrics—including demographic parity and equalized odds enable detection of spurious associations and improve transparency [34, 35]. Causal modeling approaches aim to address confounding by incorporating explicit assumptions about data-generating mechanisms. Techniques such as propensity score adjustment, causal inference frameworks, and Bayesian networks have been applied to disentangle true causal relationships from dataset-specific correlations [29, 36]. While these approaches enhance interpretability and support bias detection, their effectiveness depends on data quality, appropriate model assumptions, and the availability of relevant covariates, and should therefore be considered complementary rather than standalone solutions.

Mitigation strategies in AI/ML-based AMR prediction can be conceptualized across four interrelated domains: (1) data rebalancing and sampling strategies; (2) structure-aware modeling and data partitioning; (3) robust and context-aware evaluation frameworks; and (4) explainability and causal inference approaches. No single strategy fully resolves bias or heterogeneity. Effective mitigation requires an integrated, multi-layered approach spanning the entire machine learning pipeline [6, 11]. The following narrative integrates the key findings across data domains, modeling approaches, performance ranges, validation strategies, and limitations identified in the included studies.

Across the five major data domains identified in this review, consistent patterns of performance and limitation are evident. WGS-based models — predominantly employing random forests, gradient boosting, convolutional neural networks, or deep neural networks — reported the highest discrimination performance, with AUROC values typically ranging from 0.80 to 0.97. However, this strong performance was almost exclusively observed under internal validation conditions. Where external validation was reported, accuracy declined substantially, indicating that WGS-based models remain sensitive to population-level genomic diversity, sequencing platform differences, and bioinformatics pipeline variability across institutions. The assumption that resistance genotypes translate uniformly to resistance phenotypes across settings was frequently violated [17, 27].

AST-based models, using algorithms including random forests, support vector machines, and gradient boosting, achieved AUROC values in the range of 0.65 to 0.90. These studies were predominantly evaluated on single-centre internal datasets and were subject to laboratory protocol variability, breakpoint definition differences between CLSI and EUCAST standards, and class imbalance in resistance phenotypes — all of which constrained generalisability. EHR-based models, employing logistic regression, random forests, and gradient boosting, reported AUROC values between 0.60 and 0.85, with most relying on internal validation only. Missing data patterns, variability in clinical coding systems, and healthcare access disparities introduced additional heterogeneity that further reduced model transportability [23, 24].

Surveillance-based time-series models achieved AUROC values of approximately 0.70 to 0.92 but were limited by regional reporting inconsistencies and infrequent external validation. MALDI-TOF mass spectrometry-based models exhibited the widest performance range, from 0.48 to 0.85, reflecting substantial sensitivity to instrument calibration, sample preparation protocols, and spectral preprocessing procedures across laboratories [18, 25]. Across all domains, the dominant methodological pattern was the prioritisation of internal performance optimisation over external generalisability, fairness evaluation, or prospective validation — a pattern that represents the field’s most pervasive limitation.

## Proposed framework: heterogeneity mitigation framework

To address the multifaceted sources of data heterogeneity and algorithmic bias identified in this review, we propose a four-layer Heterogeneity Mitigation Framework spanning the full machine learning pipeline, from data generation to real-world deployment. The framework integrates data standardization, bias-aware modeling, robust evaluation, and governance mechanisms, reflecting current best practices in AMR research and computational medicine [6, 37].

Multiple dimensions of heterogeneity—including data modality, laboratory practices, population characteristics, study design, and modeling approaches—interact to produce distinct but interrelated forms of bias, such as sampling bias, label bias, structural confounding, and clinical context bias. These interactions collectively influence model performance, fairness, and generalizability. Importantly, mitigation strategies operate across all dimensions, emphasizing the need for an integrated, end-to-end approach rather than isolated interventions.

### Layer 1: Data and infrastructure

Bias mitigation begins at the data level, where heterogeneity is most pronounced. Standardization of AST protocols including harmonization of laboratory procedures and breakpoint systems is critical to reduce label inconsistency and improve comparability across datasets [38]. Equally, standardized genomic quality control pipelines for WGS including consistent assembly, annotation, and resistance gene detection workflows are necessary to minimize inter-laboratory variability [27, 39]. Addressing global sampling imbalance requires expanding surveillance and data collection in underrepresented regions, particularly LMICs, to improve model generalizability across diverse resistance ecologies [13]. Development of well-curated benchmarking datasets with balanced representation across species, geography, and time further supports reproducibility and fair model comparison [27, 39].

### Layer 2: Modeling approaches

At the modeling stage, mitigation strategies focus on improving robustness while reducing reliance on spurious correlations in heterogeneous data. Robust algorithms including ensemble methods such as random forests and gradient boosting have demonstrated resilience to noisy and heterogeneous datasets [21]. Bias-aware sample weighting can further improve fairness by adjusting for underrepresented groups, including geographic regions, pathogen types, and resistance classes. Structure-aware modeling approaches are particularly important in AMR prediction; incorporating bacterial population structure or phylogenetic information helps prevent models from exploiting lineage as a proxy for resistance, thereby improving biological validity and generalizability [17]. Strategies for handling class imbalance such as resampling and cost-sensitive learning can enhance detection of rare resistance phenotypes, although their effectiveness remains context-dependent [6].

### Layer 3: Evaluation standards

Robust evaluation is essential to ensure that models generalize beyond their training environment and provide clinically meaningful predictions. External validation using independent datasets and temporal validation across different time periods are critical for assessing real-world performance and identifying overfitting [3]. Beyond traditional performance metrics such as AUROC, evaluation should incorporate calibration, subgroup performance, and fairness-oriented metrics to assess reliability across diverse populations [11]. Benchmarking against clinical baselines such as antibiograms or clinician decision-making is also necessary to confirm that AI/ML models provide meaningful improvements over existing practice [40].

### Layer 4: Governance and implementation

Effective deployment of AI/ML-based AMR prediction models requires alignment with clinical workflows, ethical standards, and regulatory frameworks. Integration into antimicrobial stewardship (AMS) programs ensures that predictive models support, rather than replace, clinical decision-making, particularly in empiric therapy selection [41, 42]. Ethical considerations—including fairness, transparency, and accountability—are essential to prevent reinforcement of existing healthcare inequities [43]. Ongoing monitoring of model performance is required to detect degradation due to temporal changes in resistance patterns and clinical practices. Adherence to data governance and privacy frameworks is also necessary for responsible and sustainable AI deployment in healthcare [44].

The proposed framework conceptualizes bias mitigation in AI/ML-based AMR prediction as a continuous, multi-layered process. Data-level interventions address representativeness and measurement validity; modeling approaches reduce structural and algorithmic bias; evaluation strategies ensure robustness and generalizability; and governance mechanisms enable safe and equitable deployment. Critically, these layers are interdependent—limitations in data quality and representation cannot be fully corrected through downstream modeling or evaluation alone. Effective AMR prediction systems therefore require an end-to-end design approach that integrates epidemiological, microbiological, and computational perspectives. The pipeline-stage mapping of heterogeneity, bias, and mitigation strategies synthesised from this review is described narratively in the following section.

The pipeline-stage mapping reveals that distinct heterogeneity sources and bias types interact at each modelling stage to shape downstream reliability. During data collection, geographic imbalance and LMIC underrepresentation produce sampling bias; multi-centre datasets and inclusive sampling protocols are the primary correctives. At the labelling stage, CLSI/EUCAST breakpoint variability generates label inconsistency and misclassification risk; standardisation and the use of raw MIC values alongside categorical labels are required. Feature engineering is affected by EHR coding variability and missing data, introducing measurement bias; systematic preprocessing and validation pipelines address this. During model training, class imbalance across resistance phenotypes generates algorithmic bias and unequal predictive performance across resistance classes; resampling, cost-sensitive learning, and structure-aware partitioning are relevant. Model validation is undermined by absent or inadequate external datasets, producing evaluation bias that inflates reported performance; mandatory external and temporal validation is the necessary response. At deployment, temporal drift in resistance epidemiology and evolving clinical practice cause progressive performance degradation; continuous post-deployment monitoring and scheduled model updating are required. These stages are interdependent: weaknesses in data collection and labelling propagate through all downstream steps and cannot be corrected by modelling or evaluation strategies alone.

## Discussion

### Summary of key findings

This systematic review with descriptive synthesis demonstrates that AI/ML approaches for AMR prediction consistently achieve strong internal performance, frequently reporting high discrimination metrics such as area under the receiver operating characteristic curve (AUROC). However, this apparent success is substantially constrained by pervasive data heterogeneity and multiple forms of algorithmic bias that collectively limit real-world applicability and clinical translation [3, 37].

A central finding is that model performance is highly context-dependent. Many studies rely on internally consistent datasets, enabling models to implicitly learn local epidemiological patterns, institutional practices, or population structure rather than generalizable biological mechanisms. As a result, models often perform well under controlled conditions but fail to maintain accuracy when applied across different geographic regions, healthcare settings, or time periods [4, 9].

Data heterogeneity emerged as a multi-dimensional challenge encompassing variability in data modalities, laboratory protocols, population composition, and temporal and geographic distribution. These interacting dimensions complicate data integration, reduce reproducibility, and contribute to inconsistent model performance. In particular, the dominance of high-income country datasets and hospital-based populations introduces structural imbalances that further limit generalizability and equity.

Algorithmic bias was similarly pervasive, with sampling bias, label inconsistency, structural confounding, and clinical context bias identified as major contributors to model fragility. These biases often cause models to exploit spurious correlations such as phylogenetic structure, institutional prevalence, or documentation patterns rather than learning causal determinants of resistance [21, 29]. Consequently, high internal accuracy frequently masks poor performance in external or real-world settings.

Importantly, external validation remains limited across the literature. Where implemented, it consistently reveals reduced predictive performance compared to internal validation, highlighting the extent to which current evaluation practices overestimate model reliability [5, 6]. This gap underscores a broader methodological issue in AMR machine learning: the prioritization of predictive accuracy over robustness, fairness, and generalizability. Taken together, these findings indicate that the primary constraint in AI/ML-based AMR prediction is not algorithmic capability, but the quality, structure, and representativeness of the underlying data.

### Interpretation and implications

The findings of this review do not merely confirm that data heterogeneity and algorithmic bias limit AI/ML performance in AMR prediction — a point now well-established in the literature. The more analytically significant contribution is the characterisation of why these problems persist despite being widely recognised, and what structural mechanisms sustain them across successive generations of studies. Three interconnected mechanisms explain this persistence, and understanding them is prerequisite to addressing them effectively.

The first mechanism is structural: the incentive environment of academic machine learning research systematically rewards demonstration of high internal performance over external generalisability. Models are evaluated on held-out portions of the same dataset on which they were trained, under conditions that preserve the local epidemiological, institutional, and demographic characteristics of the training data. This is not merely methodological oversight; it reflects a publication culture in which AUROC improvements are the primary marker of contribution, and in which external validation — which consistently reduces apparent performance — is optional. The result is a field that produces progressively more accurate descriptions of its own training data without proportionate advances in real-world deployability. Data heterogeneity is not the fundamental cause of this failure; the fundamental cause is an evaluation norm that makes heterogeneity invisible until the moment of deployment.

The second mechanism is infrastructural: the data ecosystems required for robust, generalisable AMR prediction do not yet exist at the scale or quality needed, and the actors with capacity to build them — national public health agencies, international surveillance bodies, hospital networks — face governance, legal, and resource constraints that impede data sharing even where the scientific case for it is unambiguous. This review consistently found that the most geographically representative studies were those embedded in established international surveillance infrastructure, not those conducted by individual research groups. Individual investigators cannot resolve a collective action problem in data infrastructure; this is a policy and governance challenge, not primarily a technical one. The persistence of sampling bias and geographic imbalance in the literature therefore reflects not scientific ignorance of the problem, but the absence of coordinated institutional mechanisms for addressing it [28, 30].

The third mechanism is epistemic: the way the problem is framed in the literature systematically obscures its structural nature. When heterogeneity is framed as a data quality problem, the implied solution is better data collection — a technical fix that leaves structural incentives and governance gaps intact. When bias is framed as an algorithmic problem, the implied solution is better algorithms — which similarly deflects attention from the data ecosystem conditions that generate bias regardless of model architecture. This review’s analysis suggests that reframing is itself a necessary intervention: the problem is not primarily technical but institutional, and the solutions — governance reforms, shared infrastructure, binding reporting standards, policy-level investment in LMIC surveillance — are correspondingly institutional in nature. Models that perform inconsistently across populations risk reinforcing existing health disparities in settings where AMR burden is highest but data representation is lowest [13, 43, 44].

Understanding these three mechanisms — incentive misalignment, infrastructure deficit, and epistemic framing — also explains why previous technical interventions have had limited impact. Resampling methods, fairness-aware algorithms, and improved validation protocols are all necessary, but none addresses the conditions that generate the problems in the first place. The Heterogeneity Mitigation Framework proposed in this study is designed with this analysis in mind: its governance and infrastructure layers (Layers 1 and 4) are explicitly intended to address the structural and institutional dimensions of the problem, not merely the algorithmic ones. If validated, the framework’s primary value lies not in providing new algorithms but in providing an integrated account of what needs to change and at which level of the system each intervention is most appropriately directed.

### AI in context: comparison with conventional AMR approaches

To contextualise the limitations identified in this review, it is important to examine how conventional, non-machine-learning AMR approaches handle the same sources of variability. Traditional antimicrobial susceptibility testing, governed by CLSI and EUCAST breakpoint standards, provides a standardised resistance classification framework grounded in pharmacokinetic, pharmacodynamic, and outcomes data. This standardisation substantially reduces label inconsistency relative to unstandardised ML training datasets. However, it does not eliminate inter-laboratory variability: differences in testing methodology, inoculum preparation, incubation conditions, and the timing of breakpoint updates continue to introduce residual measurement heterogeneity [22, 38]. Discordant results between CLSI and EUCAST classifications for the same organism-antibiotic combination remain a documented source of uncertainty that affects both conventional reporting and ML training labels equally [26].

International AMR surveillance frameworks — including the WHO Global Antimicrobial Resistance and Use Surveillance System (GLASS) and the European Antimicrobial Resistance Surveillance Network (EARS-Net) — have confronted geographic and demographic imbalances comparable to those affecting ML models. Despite sustained investment, surveillance coverage remains systematically skewed toward high-income settings, with LMICs substantially underrepresented relative to their AMR burden. This structural imbalance predates the use of machine learning in AMR and reflects broader systemic inequities in global health infrastructure. The critical distinction is that ML models can silently amplify these pre-existing imbalances through training, without the transparent coverage gap reporting that characterises established surveillance systems. Conventional epidemiological prediction models — such as logistic regression-based antibiograms, pharmacokinetic/pharmacodynamic targets, and Bayesian infection models — share the generalisability limitations of ML models when applied outside their derivation setting, but are generally more interpretable, more readily understood by clinicians, and less prone to overfitting [40, 42].

AI and ML approaches are therefore best understood as complements to, rather than replacements for, conventional AMR prediction and surveillance. Their primary value lies in their capacity to detect complex, non-linear associations and to integrate heterogeneous data modalities at scale — capabilities that exceed those of conventional statistical models. However, this added complexity comes with added interpretability costs, greater susceptibility to training data biases, and greater dependence on data quality and representativeness. A pragmatic integration — in which ML models augment conventional AST results, antibiogram-guided empiric therapy, and surveillance-based trend analysis rather than substituting them — is more likely to deliver reliable clinical utility than a wholesale replacement paradigm.

### Localised models, federated learning, and context-specific deployment

The heterogeneity documented in this review raises a fundamental strategic question: whether the field should continue to pursue universal predictive models trained on large, globally aggregated datasets, or whether the ecological and institutional diversity of AMR settings makes locally adapted models more clinically appropriate. The evidence synthesised here strongly supports reconsideration of the universal model paradigm. Resistance patterns, pathogen distributions, prescribing practices, patient demographics, and data infrastructures differ substantially across geographic regions, hospital types, and healthcare systems. A model trained predominantly on high-income, hospital-based data from a limited set of countries is unlikely to perform reliably across LMICs, primary care settings, or regions with distinct resistance ecologies — a pattern consistently observed in the external validation literature [4, 13].

Localised dataset development — using institution- or region-specific AST, genomic, and EHR data — offers a practically grounded alternative. Models trained on local data capture the actual resistance ecology, prescribing culture, and population characteristics of the setting in which they will be deployed. Local antibiograms, which are already widely used in clinical practice and represent an implicit form of localisation, provide a useful analogy: their effectiveness for guiding empiric therapy within their derivation institution is well established, precisely because they reflect local epidemiology [40]. However, purely localised approaches are constrained by smaller sample sizes, reduced capacity to detect rare resistance phenotypes, and limited ability to anticipate emerging resistance trends originating elsewhere before they become locally prevalent.

Hospital-specific model development represents a particularly promising and practically grounded form of localisation. A hospital-specific model is trained exclusively on data generated within a single institution — its own AST records, genomic sequences from its clinical microbiology laboratory, EHR data reflecting its patient population and prescribing culture, and its own temporal resistance trends. Such models can, in principle, capture institution-level idiosyncrasies that no multi-site model would detect: the resistance ecology of a specific ICU, the effect of local antimicrobial stewardship interventions on resistance prevalence, or the patient-mix characteristics of a tertiary referral centre versus a district hospital. The practical feasibility of hospital-specific model development has been demonstrated in antimicrobial stewardship contexts, where institution-level machine learning models have been used to guide empiric prescribing with reasonable accuracy [40, 42]. The principal challenge is that hospital-specific models require sustained local data infrastructure, regular retraining as resistance patterns evolve, and clinical governance mechanisms for ongoing monitoring — resource requirements that may be feasible in well-resourced tertiary centres but are unlikely to be sustainable in primary care settings or LMIC hospitals without targeted investment and capacity building.

Transfer learning and federated learning architectures offer promising technical pathways that address this tension. Transfer learning enables a model pre-trained on a large, data-rich source dataset to be fine-tuned using a smaller, locally specific target dataset, combining the representational capacity of the former with the contextual relevance of the latter [41]. Federated learning enables model training across multiple institutions or geographies without centralising patient data, thereby learning from diverse resistance ecologies while respecting data governance requirements and preserving privacy. Early applications of federated learning in clinical microbiology and infectious disease suggest that federated models can approach the performance of centralised models while reducing the sampling biases inherent in single-site datasets. Reinforcement learning approaches, which enable adaptive model behaviour in response to feedback, have theoretical relevance for tracking resistance evolution in real time, though robust empirical evidence for this application in AMR prediction specifically remains nascent and claims of benefit should be made with caution.

The implication for research and implementation strategy is that context-specific deployment — guided by local epidemiology, institution-level data, and iterative model updating — is likely to provide greater clinical utility than the pursuit of a single universal model. A consistent methodological framework for how local models should be built, validated, and updated, combined with active publication of locally derived datasets to enable external validation and comparison, may offer more durable public health value than any individual predictive model.

### Standardised frameworks and evaluation strategies as the primary goal

A recurring theme across the evidence synthesised in this review is that the methodological and evaluation standards applied to AI/ML-based AMR prediction are inconsistent, incompletely reported, and frequently inadequate for establishing real-world reliability. No consensus exists on what constitutes a valid external validation dataset, how performance should be disaggregated across subgroups, or what threshold of external performance justifies clinical deployment. This absence of shared standards means that published performance metrics cannot be meaningfully compared across studies, and that clinicians and policymakers lack the information needed to assess whether a given model is fit for their context [36, 44].

In this context, the development and adoption of standardised reporting frameworks — analogous to TRIPOD for clinical prediction models, or CONSORT for clinical trials — represents a higher-priority intervention than further algorithmic innovation. A reporting standard for AI/ML-based AMR prediction studies should require, at minimum: transparent description of training data provenance, size, and geographic coverage; reporting of class balance across resistance phenotypes; description of validation strategy including whether external or temporal validation was conducted; subgroup performance disaggregated by pathogen, antibiotic class, geography, and healthcare setting; and fairness metrics assessing differential performance across demographic or geographic groups. Adoption of FAIR data principles — ensuring that datasets are Findable, Accessible, Interoperable, and Reusable — for AMR datasets shared between institutions and jurisdictions would further accelerate reproducibility and independent validation [39].

The Heterogeneity Mitigation Framework proposed in this study is intended to contribute to this standardisation agenda. By organising mitigation strategies across four pipeline stages — data and infrastructure, modelling approaches, evaluation standards, and governance — it provides a conceptual scaffold for systematic, end-to-end quality assurance in AMR model development. As noted in the Limitations, the framework has not yet been empirically validated. Its adoption should be accompanied by prospective evaluation of its impact on external validation performance, fairness, and clinical utility across diverse settings.

### Future research priorities

The heterogeneity and bias identified in this review define a concrete, prioritised research agenda. Five domains of action are most urgently needed. First, globally representative AMR datasets must be developed and actively published. Current evidence is heavily concentrated in high-income, hospital-based settings; this cannot be corrected by modelling choices. Coordinated investment in surveillance infrastructure, genomic sequencing capacity, and data sharing mechanisms in LMICs — coupled with open-access publication under FAIR principles — is essential. Multicenter collaborations such as those coordinated through GLASS provide an existing infrastructure that could be leveraged and expanded for AI-oriented data collection [28].

Second, harmonisation of laboratory and reporting standards is a prerequisite for cross-study comparability. Convergence between CLSI and EUCAST breakpoint systems, or consistent reporting of raw MIC values alongside categorical interpretations, would substantially reduce label inconsistency. Standardised bioinformatics pipelines for WGS-based resistance prediction — with transparent documentation of assembly, annotation, and resistance gene detection methods — should be adopted as reporting norms [27, 39].

Third, external and temporal validation must become mandatory minimum reporting requirements rather than optional additions. Journals and funding bodies in this space should establish explicit expectations that AI/ML AMR prediction studies include validation on at least one independent external dataset drawn from a different institution, geography, or time period than the training data. Reporting of performance disaggregated by pathogen, antibiotic class, geographic setting, and patient subgroup should accompany all external validation results.

Fourth, fairness auditing should become standard practice. Models that perform well on aggregate metrics but poorly for specific subpopulations — particularly LMIC patients, specific resistance phenotypes, or under-represented pathogen species — may cause harm if deployed without awareness of these limitations. Fairness-aware evaluation metrics, analogous to those used in other clinical AI applications, should be reported alongside discrimination metrics [11, 43].

Fifth, prospective clinical validation is urgently needed. The overwhelming majority of studies in this review are retrospective in design; models trained on historical data and evaluated under controlled conditions cannot be assumed to perform reliably in prospective clinical use, where data arrives sequentially, clinician behaviour adapts to model outputs, and resistance patterns evolve. Prospective validation studies embedded in antimicrobial stewardship programmes, with prespecified performance thresholds and clinical outcome endpoints, represent the necessary bridge between research and implementation [41, 42].

### Limitations

This study has several limitations warranting consideration. First, this systematic review employed descriptive synthesis rather than formal meta-analysis. No pooled estimates of model performance or formal assessment of statistical heterogeneity across studies were produced, as the degree of methodological, clinical, and outcome heterogeneity across included studies precluded meaningful statistical aggregation. Consequently, findings are presented as structured qualitative and descriptive syntheses.

Second, the review is dependent on the quality and reporting of included studies. Variability in study design, data sources, outcome definitions, and evaluation metrics limited direct comparability. Inconsistent reporting of validation strategies, preprocessing methods, and bias mitigation techniques may have led to underestimation or overestimation of certain methodological challenges.

Third, publication bias may be present, as studies reporting higher predictive performance or novel methodologies are more likely to be published, potentially yielding an overly optimistic representation of AI performance in AMR prediction. Exclusion of grey literature and non-English publications may also have limited comprehensiveness.

Fourth, the rapidly evolving nature of AI and AMR research means that emerging methodologies, datasets, and real-world implementation studies may not be fully captured within the review timeframe.

Fifth, geographical imbalance in the included literature is a substantive limitation of this review. As documented in the Results, the overwhelming majority of included studies were conducted in high-income country settings, with LMICs substantially underrepresented relative to their global AMR burden. This geographical skew directly limits the scope of conclusions that can be drawn: the heterogeneity sources, bias patterns, and mitigation strategies identified in this review disproportionately reflect data ecosystems, laboratory infrastructures, and healthcare contexts found in high-income settings. It cannot be assumed that the same patterns, priorities, or mitigation strategies apply equally in LMIC contexts, where resistance ecologies, data quality, and resource constraints differ substantially. Future systematic reviews with broader geographic representation — including purposive searching in regional databases and non-English literature — are needed to address this gap.

Sixth, the retrospective design of the overwhelming majority of included studies represents a limitation both of the primary literature and, consequently, of this review. Retrospective studies evaluate model performance on data that was collected independently of the model’s deployment, under conditions that may not reflect the distributional shifts, feedback loops, and evolving resistance patterns characteristic of real-world clinical use. The findings of this review therefore primarily characterise AI/ML performance under historically controlled conditions rather than under prospective deployment. This distinction is clinically important: a model that performs well retrospectively may behave differently when clinician prescribing adapts in response to model outputs, when resistance patterns shift, or when patient case-mix evolves. The absence of prospective validation studies in the included literature is itself a significant gap in the evidence base, not merely a limitation of this review’s scope.

Seventh, the limited availability of external validation data is a constraint that affects both the primary studies included in this review and the conclusions that can be drawn from them. As noted throughout the Results and Discussion, most included studies relied on internal validation only; where external validation was conducted, it consistently revealed lower performance than internal estimates. This review is therefore largely unable to characterise the true external generalisability of the AI/ML approaches examined, because the primary evidence base does not supply this information at sufficient scale or consistency. Conclusions about model robustness and generalisability are accordingly grounded in the pattern of performance degradation observed in the minority of studies that did conduct external validation, supplemented by theoretical reasoning, rather than in a comprehensive empirical assessment of cross-setting performance.

Finally, while this study proposes a Heterogeneity Mitigation Framework, it has not yet been empirically validated. Its effectiveness will depend on future application and evaluation across diverse datasets and clinical settings. It should therefore be considered a conceptual guide rather than a definitive solution.

## Conclusions

This systematic review demonstrates that AI/ML-based approaches to AMR prediction can achieve strong predictive performance under controlled conditions, but that performance is neither stable nor generalizable in the absence of high-quality, representative, and harmonized data. The primary constraint is not algorithmic sophistication but the quality, structure, and diversity of the underlying training data, alongside systematic biases embedded therein [3, 6].

The findings highlight that strong internal model performance does not reliably translate into consistent performance across diverse populations, healthcare settings, and time periods. Variability in data modalities, laboratory practices, population representation, and evaluation strategies introduces systematic challenges that reduce reproducibility and external validity. These issues are further compounded by limited use of external validation and the underrepresentation of LMICs in existing datasets, despite their bearing the highest burden of AMR [13].

Addressing these challenges requires specific, concrete actions rather than general exhortations toward improvement. The research community should: (1) establish globally representative, multi-centre AMR datasets with open-access publication and FAIR data principles, prioritising LMIC settings; (2) harmonize AST protocols and breakpoint systems, including convergence between CLSI and EUCAST standards, and adopt standardized WGS bioinformatics pipelines; (3) mandate external and temporal validation as minimum reporting requirements, with subgroup performance disaggregated by geography, pathogen, and clinical context; (4) implement fairness auditing to detect and report differential performance across demographic and geographic groups; and (5) pursue prospective clinical validation before deployment in antimicrobial stewardship programmes. The Heterogeneity Mitigation Framework proposed here provides a structured, pipeline-spanning approach to these priorities.

Multicenter collaborations and federated learning architectures offer practical pathways for building models that are locally relevant without sacrificing the scale needed for robust generalisation. Rather than pursuing a single universal model, the field may derive greater clinical utility from developing locally adapted models governed by shared methodological standards and transparent reporting frameworks. Interdisciplinary collaboration among clinicians, microbiologists, data scientists, epidemiologists, and policymakers is essential throughout this process.

The Heterogeneity Mitigation Framework proposed in this study has not yet been empirically validated and should be regarded as a conceptual guide requiring prospective testing rather than a proven solution. Ultimately, the success of AI in AMR prediction will depend not on algorithmic novelty alone, but on the scientific community’s commitment to data quality, methodological rigour, representativeness, and equitable design — commitments that this review finds are inconsistently upheld in the current literature.

## Data Availability

All data analyzed in this study are derived from previously published articles, which are cited within the manuscript. No new datasets were generated. The extracted data supporting the findings of this study are available from the corresponding author upon reasonable request.
